# In vitro efficacy of Er:YAG laser-activated irrigation versus passive ultrasonic irrigation and sonic-powered irrigation for treating multispecies biofilms in artificial grooves and dentinal tubules: an SEM and CLSM study

**DOI:** 10.1186/s12903-024-04042-x

**Published:** 2024-02-22

**Authors:** Pingping Bao, He Liu, Lan Yang, Lulu Zhang, Liwei Yang, Nannan Xiao, Jing Shen, Jiayin Deng, Ya Shen

**Affiliations:** 1 School and Hospital of Stomatology, Tianjin Medical University, Tianjin, 300070 China; 2 Department of Endodontics, School of Medicine, Tianjin Stomatological Hospital, Nankai University, Tianjin, China; 3 Tianjin Key Laboratory of Oral and Maxillofacial Function Reconstruction, Tianjin, 300041 China; 4 Department of Oral Biological & Medical Sciences, Faculty of Dentistry, University of British Columbia, 2199 Wesbrook Mall, Vancouver, BC V6T 1Z3 Canada; 5 Hangzhou Stomatological Hospital, Hangzhou, China; 6 State Key Laboratory of Medicinal Chemical Biology, Nankai University, Tianjin, China

**Keywords:** Artificial grooves, Biofilm, Root canal irrigation, Laser, Ultrasonic, Confocal laser scanning microscopy, Scanning electron microscopy

## Abstract

**Background:**

Multispecies biofilms located in the anatomical intricacies of the root canal system remain the greatest challenge in root canal disinfection. The efficacy of Er:YAG laser-activated irrigation techniques for treating multispecies biofilms in these hard-to-reach areas has not been proved. The objective of this laboratory study was to evaluate the effectiveness of two Er:YAG laser-activated irrigation techniques, namely, photon-induced photoacoustic streaming (PIPS) and shock wave-enhanced emission photoacoustic streaming (SWEEPS), in treating multispecies biofilms within apical artificial grooves and dentinal tubules, in comparison with conventional needle irrigation (CNI), passive ultrasonic irrigation (PUI), and sonic-powered irrigation (EDDY). Two types of multispecies root canal biofilm models were established in combination with two assessment methods using scanning electron microscopy (SEM) and confocal laser scanning microscopy (CLSM) with the aim to obtain more meaningful results.

**Methods:**

Ninety extracted human single-rooted premolars were chosen for two multispecies biofilm models. Each tooth was longitudinally split into two halves. In the first model, a deep narrow groove was created in the apical segment of the canal wall. After cultivating a mixed bacterial biofilm for 4 weeks, the split halves were reassembled and subjected to five irrigation techniques: CNI, PUI, EDD, PIPS, and SWEEPS. The residual biofilms inside and outside the groove in Model 1 were analyzed using SEM. For Model 2, the specimens were split longitudinally once more to evaluate the percentage of killed bacteria in the dentinal tubules across different canal sections (apical, middle, and coronal thirds) using CLSM. One-way analysis of variance and post hoc multiple comparisons were used to assess the antibiofilm efficacy of the 5 irrigation techniques.

**Results:**

Robust biofilm growth was observed in all negative controls after 4 weeks. In Model 1, within each group, significantly fewer bacteria remained outside the groove than inside the groove (*P* < 0.05). SWEEPS, PIPS and EDDY had significantly greater biofilm removal efficacy than CNI and PUI, both from the outside and inside the groove (*P* < 0.05). Although SWEEPS was more effective than both PIPS and EDDY at removing biofilms inside the groove (*P* < 0.05), there were no significant differences among these methods outside the groove (*P* > 0.05). In Model 2, SWEEPS and EDDY exhibited superior bacterial killing efficacy within the dentinal tubules, followed by PIPS, PUI, and CNI (*P* < 0.05).

**Conclusion:**

Er:YAG laser-activated irrigation techniques, along with EDDY, demonstrated significant antibiofilm efficacy in apical artificial grooves and dentinal tubules, areas that are typically challenging to access.

## Background

Successful endodontic therapy requires elimination of endodontic biofilms through meticulous root canal disinfection methods [1]. Disinfection is challenging when bacteria are organized in multispecies matrix-enclosed communities called multispecies biofilms because of their increased resistance to antimicrobial treatment (antibiotics and disinfectants) compared to that of mono-species biofilms [2]. Root canal infections have been proven to be caused by multispecies biofilms [3] colonizing not only the canal wall but also the dentinal tubules, fins, lateral canals, isthmuses, and apical ramifications [4, 5]. Therefore, multispecies biofilms located within the anatomical intricacies of the root canal system pose a greater challenge to root canal disinfection, especially in the apical region. Hand/rotary instruments cannot be used to gain access to these areas, and the smear layer and dentine debris created by instrumentation may be pushed into and accumulate in the irregularities [6]. Therefore, irrigation, as the only way to clean these confined hard-to-reach areas [7], is expected to perform most of the cleaning and disinfection [8].

Different irrigating solutions and a variety of delivery devices have been introduced for efficient disinfection. Sodium hypochlorite (NaOCl) is the most prevalent chemical irrigant due to its exceptional antimicrobial action, particularly against bacteria organized in biofilms, and its unique tissue-dissolving capacity [8, 9]. After NaOCl, the use of a chelator such as ethylenediaminetetraacetic acid (EDTA) as a final irrigant is recommended [7]. Various agitation methods, such as sonic irrigation, passive ultrasonic irrigation (PUI) and different types of lasers, have been proposed to improve irrigation efficiency and achieve optimal spreading of irrigants throughout the root canal system for more predictable cleaning of the hard-to-reach areas [7, 10]. Compared to conventional needle irrigation (CNI), PUI and sonic-powered irrigation [EDDY (VDW, Munich, Germany)] have demonstrated superior effectiveness in detaching bacterial biofilms from the root canal walls and augment the effectiveness of NaOCl in killing bacteria and enhancing its reach into dentinal tubules [11–13]. Although ultrasonic activation has been demonstrated to be effective, it carries the risk of file fracture and potential damage to the canal walls [6]. As an alternative, the EDDY tip, characterized by its smooth and highly flexible polymer construction, is powered by a sonic scaler operating at a frequency of 6,000 Hz. This enables it to move in a three-dimensional pattern with high amplitude, suggesting its potential as a substitute for PUI in activating irrigants [14].

In recent years, Er:YAG laser-activated irrigation (LAI) techniques of photon-induced photoacoustic streaming (PIPS) and shock wave-enhanced emission photoacoustic streaming (SWEEPS), characterized by the use of a shorter pulse duration (50 /25µsec) and special laser tips placed in the pulp chamber, have been introduced as potential alternatives to CNI, PUI, and EDDY [15–20] and have been recommended for the cleaning of minimally shaped root canals [16, 17]. A number of studies have evaluated the efficacy of these Er:YAG LAI techniques for dissolving pulp tissue [15, 18] and removing smear layers [19, 20] and hard tissue debris [16, 20], demonstrating their improvement over conventional irrigation. However, their superiority in removing biofilms or killing bacteria in the root canal system has not been fully elucidated, and the evidence is still limited. Most published studies have been conducted using simple mono-species *E. faecalis* biofilm models [21–23] or artificial biofilms in synthetic root canal models [6, 24]. Only one recent study [10] developed a mature polymicrobial biofilm model by using the mesial root of permanent first molars and human saliva. To the best of our knowledge, no published study has evaluated the efficacy of the two Er:YAG LAI techniques for eradicating multispecies biofilms located within the anatomical intricacies (such as apical uninstrumented area and dentinal tubules) of the root canal system in comparison with CNI, PUI, and EDDY. Studies on the antimicrobial effect of irrigants need to use mature multispecies biofilms grown on dentine or inside root canals and need to combine at least two complementary evaluation methods [25]. Therefore, in our study, two types of multispecies root canal biofilm models were established in combination with two assessment methods, scanning electron microscopy (SEM) and confocal laser scanning microscopy (CLSM), with the aim to obtain more meaningful results. The objective of this study was to investigate the efficacy of CNI, PUI, EDDY, PIPS, and SWEEPS for biofilm removal in a standardized multispecies biofilm model with a narrow and deep artificial apical groove using SEM analysis and to kill bacteria within dentinal tubules in a multispecies dentin canal biofilm model using CLSM analysis. Our hypothesis posits that no significant difference will be observed in antibiofilm efficacy among these techniques across both models.

## Methods

**Fig. 1:**
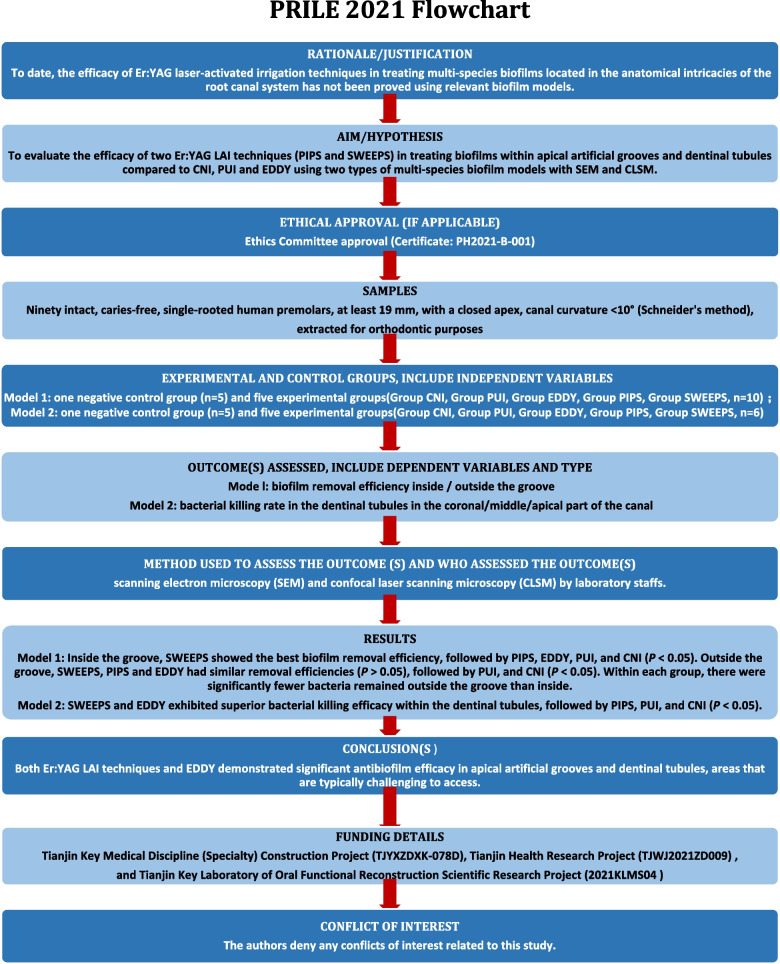
PRILE 2021 flowchart

This study was performed according to the Preferred Reporting Items for Laboratory Studies in Endodontology (PRILE) 2021 guidelines [26]. The flowchart of the PRILE 2021 trial is shown in Fig. 1. This study was approved by the Ethics Committee of Tianjin Stomatological Hospital (Certificate: PH2021-B-001). All participants provided signed informed consent, authorizing the use of their extracted premolars for research purposes. If the subjects were under 16 years of age, informed consent was obtained from their parents or legal guardians. The authors ensure that all procedures were conducted in accordance with the relevant guidelines and regulations.

### Sample size calculation

In this study, two types of biofilm models were developed and evaluated using two methods, each requiring separate sample size calculations based on our pilot study data using G*Power software v. 3.1.9.7 (Heinrich Heine University, Düsseldorf, Germany). Calculations ensuring 90% power and 5% type I error, with effect sizes of 0.7 and 0.98, indicated a minimum of 9 and 5 samples per group, respectively. The final sample sizes were increased to 10 for one test and 6 for the other per group.

### Tooth selection and preparation

**Fig. 2:**
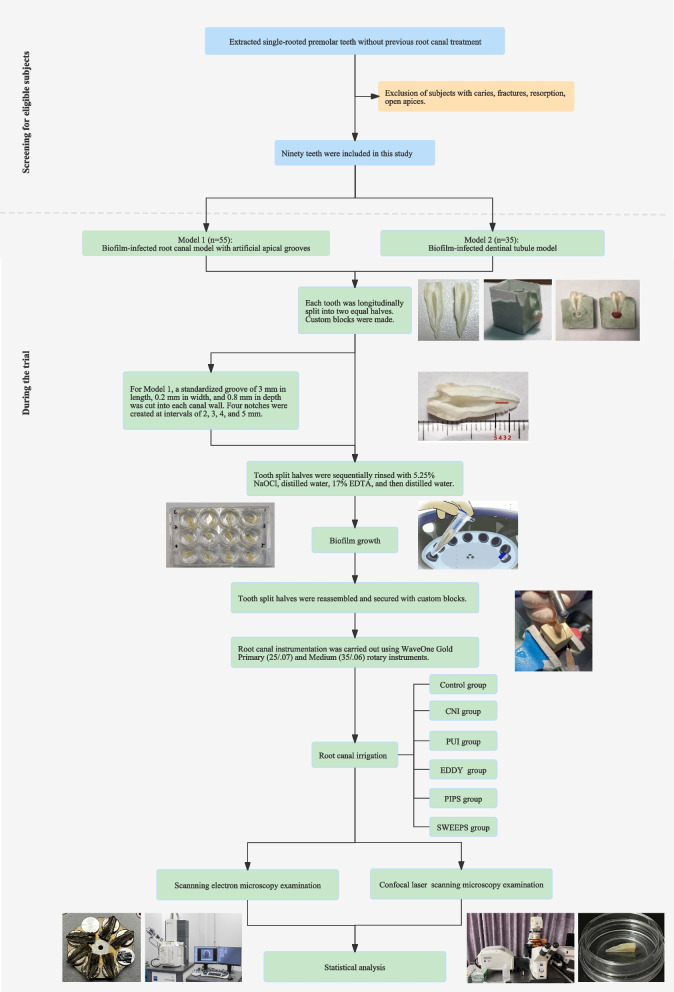
The flow diagram on the selection of eligible subjects and the conduction of the trial

Ninety intact, caries-free, single-rooted human premolars, each at least 19 mm in length with a closed apex and canal curvature < 10° (Schneider’s method), extracted for orthodontic purposes were collected. Teeth with resorptive defects, visible cracks or fractures, or previous endodontic treatment were excluded. Calculus and debris were removed from the teeth using a hand scaler. The teeth were stored at 4℃ in a 0.01% NaOCl solution until use. Figure 2 shows the flow diagram of the selection of eligible subjects and the conduction of the trial. Fifty-five teeth were randomly selected to develop a multispecies biofilm infected root canal model with an artificial apical groove (Model 1). The remaining thirty-five teeth were used to develop a multispecies biofilm infected dentinal tubules model (Model 2).

Following a previously described protocol [11], the teeth were accessed, and the reference cusps were reduced until each tooth was 19 mm in length. The working length (WL) was set at 18 mm, determined by inserting a #10 hand K-file until it was 1 mm short of the apical foramen. To standardize the initial apical geometry and prepare for bacterial contamination, the canals were instrumented using ProTaper S1 and S2 rotary NiTi instruments (Dentsply Tulsa Dental, Tulsa, OK, USA), ensuring that dentin debris was visible in the apical 4 mm portion of the S2 file.

Each tooth was then longitudinally split into two equal halves. Two grooves were created along the long axis on both the buccal and lingual surfaces of the tooth using a diamond disc under water cooling, ensuring no penetration of the root canal system. The teeth were subsequently split into two halves using a fine razor blade and a hammer. Under an operating microscope, the tooth split halves were examined, and only those teeth whose halves could be fully reassembled were included in the study.

The split halves of each tooth were reassembled and secured with a 5-mm-diameter wax ball over the root tip. Half of the split tooth and wax ball were first embedded in high-strength dental stone, followed by a further pour of dental stone covering the left half, with a thin layer of separator in between. Once the stone had set, the tooth and wax ball were removed, and the model was polished and refined. The resulting custom blocks had an apical cavity left by the wax ball, simulating periapical lesions within the alveolar bone.

For the tooth split halves designated for Model 1, a standardized groove measuring 3 mm in length, 0.2 mm in width, and 0.8 mm in depth was cut into each canal wall using a small diamond disc. The groove started 2 mm from the apex, simulating a deep, narrow fin. Four notches were created at intervals of 2, 3, 4, and 5 mm from the apex on each side, serving as reference points to identify the apical, middle, and coronal thirds of the apical groove after biofilm removal.

To eliminate the smear layer and disinfect the samples, all tooth split halves were sequentially rinsed with 5.25% NaOCl for 3 min, distilled water for 30 s, 17% EDTA for 3 min, and then distilled water twice for 30 s each.

### Biofilm growth

To cultivate the biofilm, supragingival and subgingival dental plaque was collected from the interdental spaces of molars of a healthy adult donor (BPE 0–1) using sterilized toothpicks. The collected plaque was then mixed in brain–heart infusion (BHI) broth (Becton Dickinson, MD, USA).

Model 1 The bacterial plaque suspension was adjusted to an optical density (OD) of 0.10 at 405 nm using a microplate reader (SPARK; Tecan, Männedorf, Switzerland).

This suspension was then diluted 10-fold with fresh BHI. The tooth split halves were placed in the wells of a 12-well tissue culture plate containing the diluted suspension. These tooth split halves were incubated in a BHI-bacteria suspension in an anaerobic environment (Bactron300; SHEL LAB, Cornelius, OR, USA) at 37℃ for 4 weeks. The medium was replaced with fresh BHI broth once a week.

Model 2 The bacterial plaque suspension was adjusted to an OD of 0.25 (at 405 nm) using a microplate reader and was incubated anaerobically at 37 °C for 2 days until the OD reached approximately 1. The BHI-bacteria suspension was then diluted 10-fold. The half of each tooth was placed in a sterile 1.5 mL Eppendorf tube containing the diluted BHI-bacteria suspension. The tubes were centrifuged three times at 3,500 × g for 5 min. Fresh bacterial solution was added after each centrifugation. After centrifugation, all tubes were incubated at 37 °C in BHI broth in air for 24 h to facilitate bacterial recovery. This was followed by anaerobic incubation at 37 °C for 4 weeks. The medium was replaced with fresh BHI broth once a week.

### Root canal instrumentation and irrigation

After 4 weeks of incubation, the test specimens were rinsed with sterile water for 1 min and air-dried, and then the split halves were reassembled. To prevent leakage of irrigant and bacteria along the contact area, a sealing film (Parafilm M Laboratory Film; Bemis Company, Inc., Neenah, WI, USA) was stretched and used to tightly wrap the teeth. After wrapping, the teeth were placed in their respective custom blocks.

Root canal preparation was carried out using WaveOne Gold Primary (25/0.07) and Medium (35/0.06) (Dentsply Sirona, Charlotte, NC, USA). The canal was rinsed with 1 mL of 3% NaOCl before each file and 4 mL after the second file. A total of 6 mL of NaOCl was used per canal.

The specimens from Model 1 and Model 2 were randomly divided into a control group (*n* = 5) and five experimental groups (*n* = 10 for Model 1 and *n* = 6 for Model 2), respectively. Although the WL and apical geometry of the teeth were standardized, the canal geometry (e.g., width) of the specimens still varied. Therefore, the specimens were first paired based on canal geometry and then assigned by simple coin toss. This was blind process with respect to the operator.

Just prior to the experimental irrigation/agitation procedures, all canals were irrigated with an additional 1 mL of 3% NaOCl.

- *Group CNI*: The canal was flushed with a continuous flow of 6 mL of 3% NaOCl for 90 s using a syringe and a 31-G side-vented needle (NaviTip Double Sideport; Ultradent, South Jordan, UT, USA). The needle was positioned 1 mm short of the WL and used with a gentle up-and-down movement.
- *Group PUI*: 6 mL of 3% NaOCl was continuously delivered into the access cavity and passively agitated using an ultrasonic device (P5 Newtron; Acteon Satelec, France) equipped with a K25 endodontic tip (25/0.02, Acteon Satelec). The device was set to a power level of 4, and agitation was performed for 90 s with the tip positioned 1 mm short of the WL.
- *Group EDDY*: An EDDY tip (20/0.02) (VDW, Munich, Germany) was inserted to a position 1 mm short of the WL. With the continuous delivery of 6 mL of 3% NaOCl into the access cavity, the tip was driven by an air scaler (Proxeo, W&H, Bürmoos, Austria) at a frequency of 6,000 Hz and moved up and down over a length of 4 mm.
- *Group PIPS*: An Er:YAG laser with a wavelength of 2,940 nm (Lightwalker; Fotona, Ljubljana, Slovenia) paired with a handpiece (H14, Fotona) that held a conical fiber tip (PIPS 600/9) was used to activate the irrigant. The tip was positioned at the canal entrance and operated with the recommended settings (SSP mode, 20 mJ, 15 Hz, 0.3 W, 0/0 air/water). While the tip remained stationary, the irrigant (2 mL of 3% NaOCl) was activated and continually added for 30 s. This cycle was repeated a total of three times, with a 30-s break between each interval.
- *Group SWEEPS*: The same 2,940 nm Er:YAG laser (Lightwalker, Fotona) and H14 handpiece were utilized. A specialized fiber tip (SWEEPS 600/10) was activated using the AutoSWEEPS mode (20 mJ, 15 Hz, 0.6 W, 0/0 air/water). The irrigation procedure used was identical to that used for Group PIPS.

In all groups, final irrigation was performed using 2 mL of sterilized water for 30 s, followed by 4 mL of 17% EDTA for 2 min.

### Scanning electron microscopy evaluation (Model 1)

All specimens from Model 1 were processed as follows: the teeth were disassembled, and the split halves were fixed in 2.5% glutaraldehyde for 24 h, dehydrated in a graded series of ethanol solutions, critical point-dried, coated with gold, and examined by scanning electron microscopy (SUPRA 55VP; Carl Zeiss, Oberkochen, Germany).

To avoid bias in the image acquisition, stratified random sampling was used as previously described [11]. Briefly, the sampling location was predetermined from inside the groove (apical, middle, or coronal third) at a low magnification, and then a higher magnification (1,000 ×) was used to obtain 3 sample areas (areas in the middle and adjacent areas on each side), and the 3 sample areas were increased in magnification to 2,000 × each for evaluation. Additional 3 images at 2,000 × magnification was recorded outside of the groove in the areas adjacent to the former 3 sample areas inside the groove. For each specimen (1 split half), a total of 18 standardized 2,000 × SEM images from all thirds inside and outside of the groove were acquired.

Eighteen hundred 2,000 × SEM images from 50 treated teeth were randomly coded and scored separately by 2 independent examiners. The percentage of residual biofilm bacteria was measured using Image-Pro Plus 6.0 software (Media Cybernetics, Bethesda, MD). Interobserver and intraobserver reproducibility were measured using the weighted coefficient kappa.

### Confocal laser scanning microscopy evaluation (Model 2)

After completing the instrumentation and irrigation processes, the teeth were disassembled and decoronated at the cemento-enamel junction. The root halves were then rinsed in sterile water for 1 min. Subsequently, each half was split longitudinally through the root canal into two halves, as previously described, to expose the fresh surface of the dentin tubules for CLSM examination. It was crucial to maintain constant water cooling while cutting grooves with the diamond disc to prevent heat generation, which could kill the bacteria. All specimens were then stained using the LIVE/DEAD BacLight Bacterial Viability Kit (L7012; Molecular Probes, Eugene, OR, USA) for 30 min. After staining, the specimens were rinsed with phosphate-buffered saline three times for 1 min each.

A Zeiss confocal microscope (LSM800; Carl Zeiss, Oberkochen, Germany) was used, and the excitation/emission wavelengths were set at 480/500 nm for SYTO 9 and 490/635 nm for propidium iodide. Three random locations from each third (apical, middle, and coronal) were scanned, resulting in a total of 9 images per specimen or 36 images per tooth. For each scan, fifty slices with a 2 μm step size were acquired. These scans were compiled using ZEN 2.6 software (blue edition) at a resolution of 512 × 512 pixels.

One thousand eighty CLSM images from 30 treated teeth were randomly coded and scored separately by 2 independent examiners. Imaris 9.8.2 software (Bitplane, Inc., St. Paul, MN, USA) was used to facilitate three-dimensional reconstruction of the scans and to calculate the volume ratio of red fluorescence to combined red‒green fluorescence; this ratio represents the percentage of dead bacteria relative to the total of dead and live bacteria. Interobserver and intraobserver reproducibility were also measured using the weighted coefficient kappa.

### Statistical analyses

One-way analysis of variance and post hoc multiple comparisons were used to compare the percentage of area covered with bacteria inside and outside the groove between different irrigation groups and different areas within a single group (Model 1) and the volume ratio of the red fluorescence to the combined red–green fluorescence between different treatment groups and different locations within 1 group (Model 2). All the statistical analyses were performed with SPSS Statistics 27.0 for Windows software (IBM Corp., NY, USA), and the significance level was set at 5%.

## Results

The interobserver and intraobserver reproducibility were 0.91 and 0.95, respectively.

### Biofilm removal efficiency inside and outside the groove (Model 1)

**Fig. 3:**
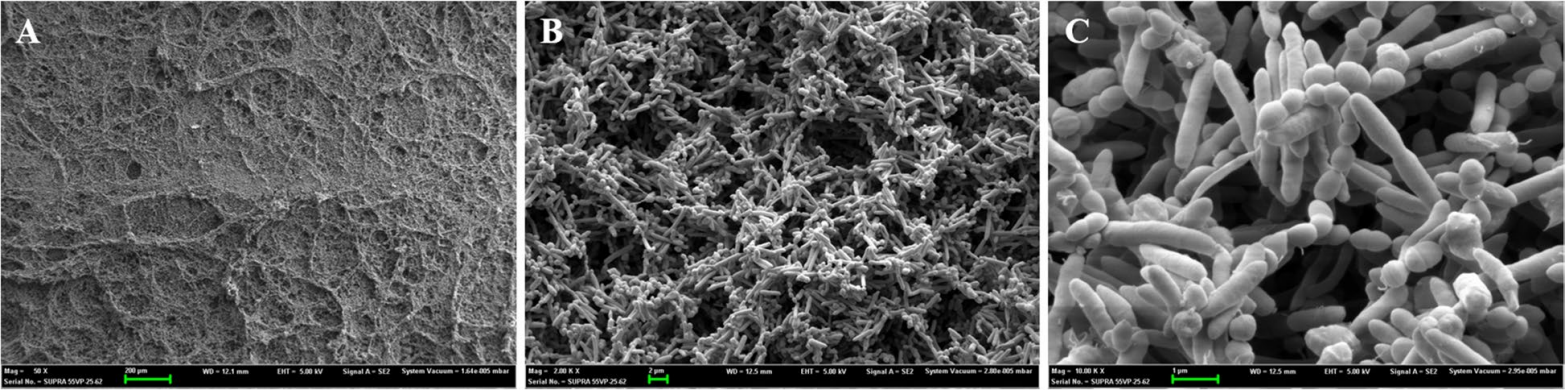
SEM images showing robust growth of multi-species biofilm covering the canal after 4-week incubation in Model 1. **A** 50 × magnification, **B** 2,000 × magnification, **C** 10,000 × magnification

**Fig. 4:**
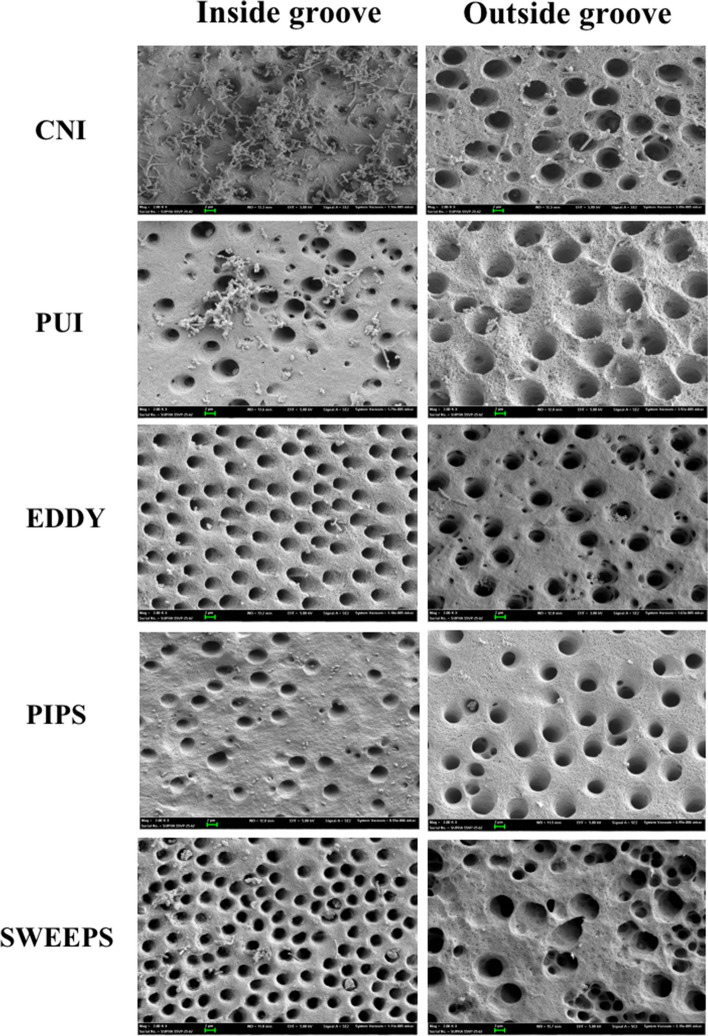
Representative SEM images showing areas inside and outside the groove after irrigation with CNI, PUI, EDDY, PIPS, SWEEPS (2,000 × magnification)

All controls showed robust and consistent growth of multispecies biofilms in the canal under SEM observation after 4 weeks of incubation. Representative SEM images of the controls are shown in Fig. 3, and the areas inside and outside the groove after treatment using different irrigation techniques are shown in Fig. 4. The average percentage of biofilm remaining inside and outside the groove in each treatment group is detailed in Table 1.

**Table 1:** Average percentages of bacteria remaining inside and outside the groove after irrigation with CNI, PUI, EDDY, PIPS and SWEEPS (Mean ± Standard Deviation)

Group	Inside groove				Outside groove			
	**Apical**	**Middle**	**Coronal**	**Total**^**a**^	**Apical**	**Middle**	**Coronal**	**Total**^**b**^
**CNI**	21.19 ± 9.55	18.14 ± 8.25	18.18 ± 9.97	19.17 ± 9.35^c^	1.48 ± 1.08	1.76 ± 1.10	1.75 ± 1.11	1.66 ± 1.10^e^
**PUI**	4.64 ± 2.40	4.12 ± 2.35	5.10 ± 2.70	4.62 ± 2.51^d^	0.79 ± 0.35	0.98 ± 0.45	0.88 ± 0.37	0.88 ± 0.40^g^
**EDDY**	2.57 ± 2.02	1.93 ± 1.82	2.01 ± 2.32	2.17 ± 2.07 ^e^	0.54 ± 0.39	0.52 ± 0.40	0.55 ± 0.43	0.54 ± 0.41^h^
**PIPS**	1.22 ± 0.77	1.11 ± 0.70	1.02 ± 0.55	1.12 ± 0.68^f^	0.57 ± 0.38	0.54 ± 0.58	0.53 ± 0.53	0.54 ± 0.50^h^
**SWEEPS**	0.85 ± 0.40	0.82 ± 0.39	0.75 ± 0.37	0.81 ± 0.39^g^	0.50 ± 0.29	0.41 ± 0.22	0.42 ± 0.24	0.44 ± 0.25^h^

Lowercase letters indicate statistically significant differences between groups (*P* < .05)

Inside the groove, SWEEPS had the greatest biofilm removal efficiency, followed by PIPS, EDDY, PUI, and CNI (*P* < 0.05). Outside the groove, SWEEPS, PIPS and EDDY had similar removal efficiencies (*P* > 0.05), followed by PUI and CNI (*P* < 0.05). In all groups, significantly fewer bacteria remained outside the groove than inside the groove (*P* < 0.05). Within each group, no statistical differences were found among the apical, middle and coronal thirds, either inside or outside the groove (*P* > 0.05).

### Efficiency of killing bacteria in dentinal tubules (Model 2)

**Fig. 5:**
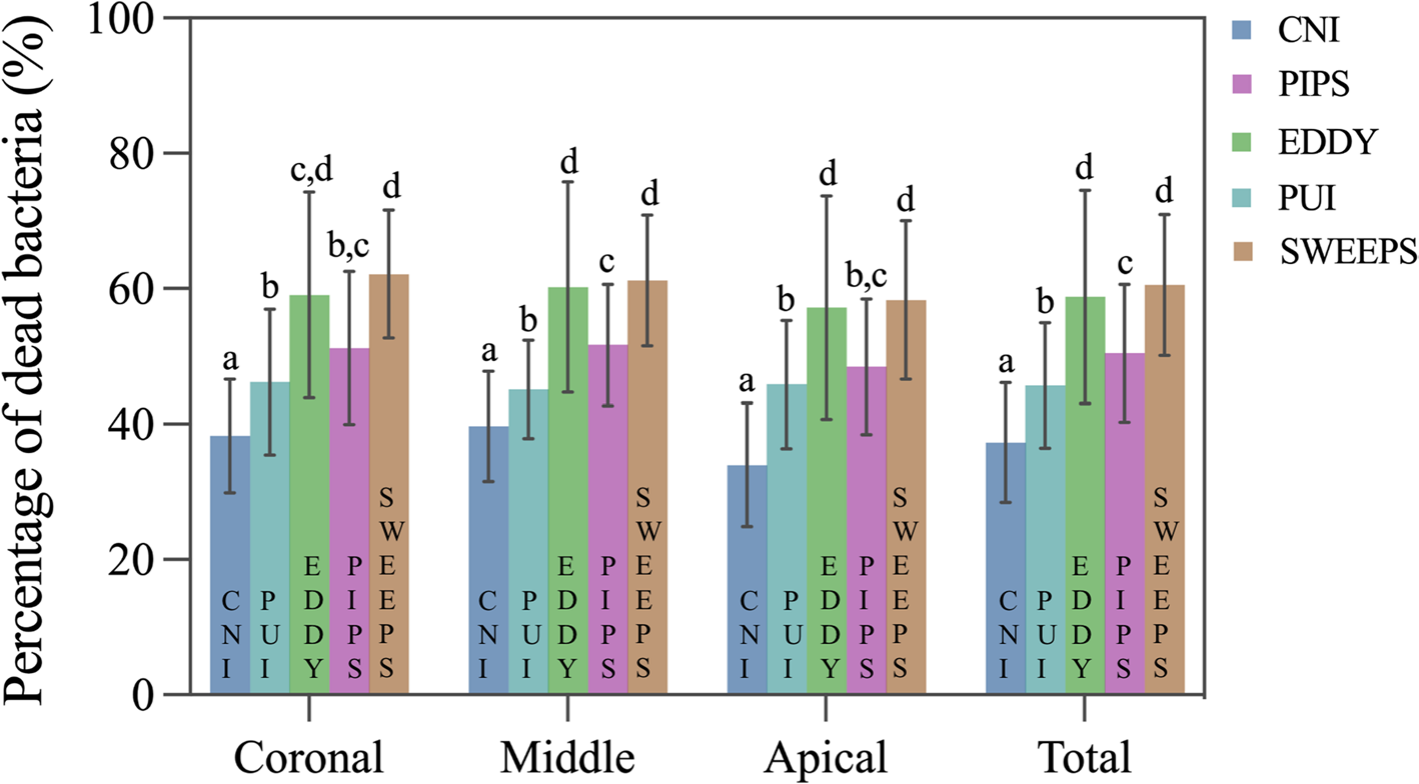
The percentage of dead bacterial in the dentinal tubules after irrigation with CNI, PUI, EDDY, PIPS and SWEEPS. Different lowercase letters indicate significant differences among various irrigation techniques within the same third of the roots (*P* < 0.05)

**Fig. 6:**
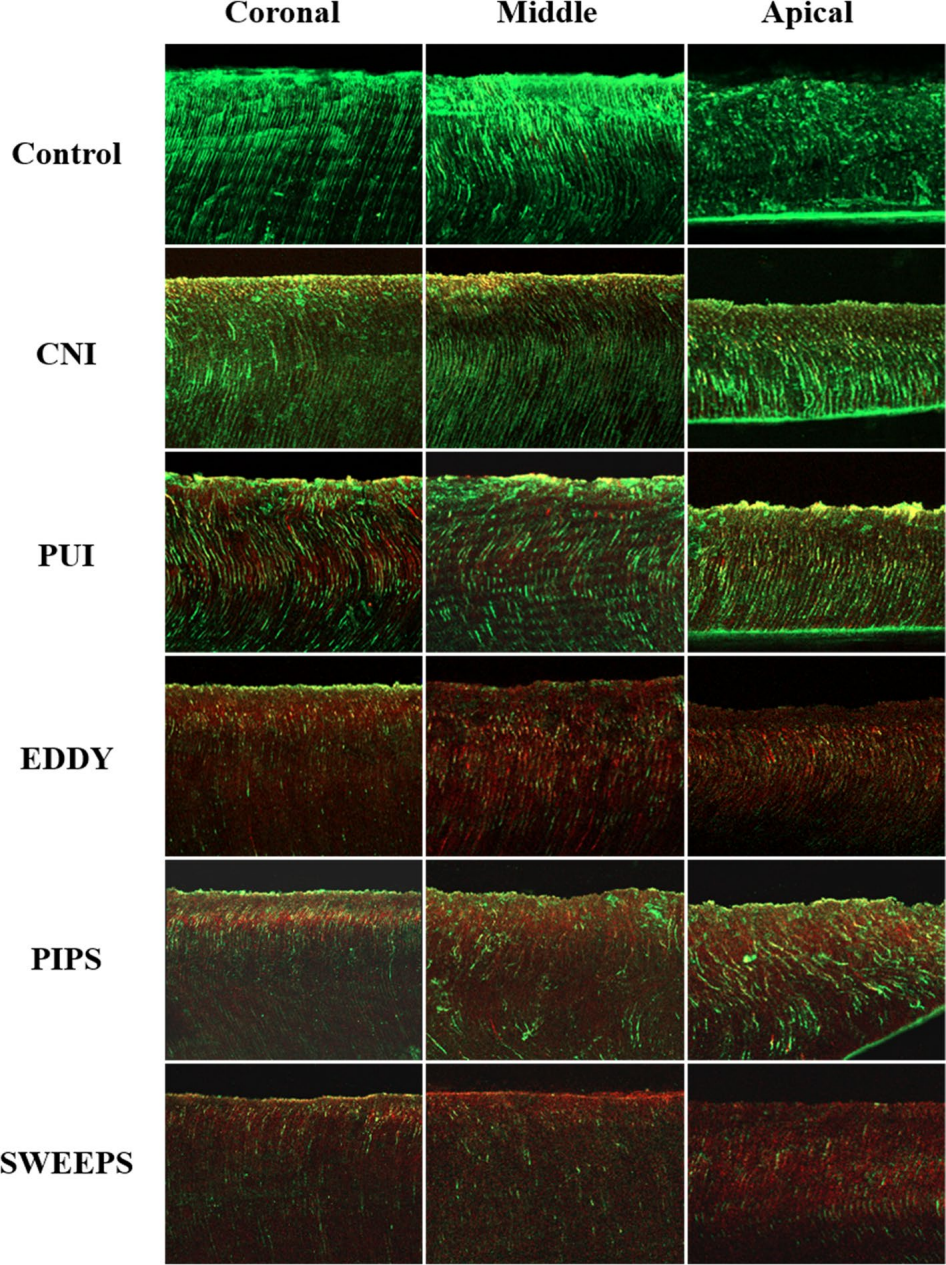
Representative CLSM images of live (green) and dead (red) bacterial cells in the dentinal tubules in the coronal, middle and apical thirds of the roots after irrigation with CNI, PUI, EDDY, PIPS and SWEEP, as well as the controls

After 4 weeks of incubation, a homogenous and dense infection from the canal side reached up almost the full length of the dentinal tubules to the dentinocemental junction were observed in all specimens. The highest mean percentage of dead bacteria was observed in the SWEEPS (60.56%) and EDDY (58.83%) groups (*P* > 0.05), followed by the PIPS (50.45%), PUI (45.71%) and CNI (37.30%) groups (*P* < 0.05) (Fig. 5). Within each group, no statistical differences were observed among the three regions (*P* > 0.05). Figure 6 shows representative CLSM images of the coronal, middle, and apical regions of each group, including the control and five experimental groups.

## Discussion

In the present study, two types of multispecies biofilm models using extracted single-rooted teeth were developed to investigate the effectiveness of 5 irrigation techniques (PIPS, SWEEPS, CNI, PUI and EDDY) in removing biofilms from apical artificial grooves and in killing bacteria within dentinal tubules. The two models were designed to simulate the complexity of root canal anatomy and to mimic natural biofilms in the root canal system. The first multispecies biofilm model with an artificial apical groove (Model 1) was used in our previous study [11], and the only difference was that in the present study, a sealing film was used to tightly wrap the teeth to provide an adequate seal between the two model halves; otherwise, irrigants and bacteria could leak along the contact area. To the best of our knowledge, the second multispecies biofilm model (Model 2) is the first to grow multispecies biofilms in dentinal tubules using full-length teeth rather than dentin blocks. Its main advantage over dentin block models is that they involve root canal geometry and can therefore be used to assess the effectiveness of antimicrobial procedures such as irrigation. Furthermore, the creation of a deep narrow groove in the main canal of Model 1 and the dentinal tubules in Model 2 facilitate the comparison of the antibiofilm efficacy of various irrigation methods in both easily accessible areas (outside the groove) and areas that are difficult to reach (inside the groove and within the dentinal tubules) within a single study.

Both SEM and CLSM are commonly used methods for confirming the presence of a biofilm and evaluating the efficacy of biofilm removal. SEM allows visualization of the morphological structures of biofilms, their amount and distribution on the dentin surface and in deeper dentin layers [13]. Although limited to two-dimensional and semiquantitative analysis, SEM is often preferred over other microscopy techniques due to its high resolution and high magnification [2]. However, the sample preparation steps can affect the original biofilm morphology and may cause the biofilm to detach from the surface. Additionally, it is important to note that the viability of microbial cells cannot be assessed using SEM [2]. CLSM is a very useful noninvasive method for studying biofilms in situ, assessing their structure and distribution without causing significant destruction [27]. Viability stains used in conjunction with CLSM allow for the determination of the viability profile of biofilm bacteria in the root canal. This enables a semiquantitative analysis, allowing for assessment of both biofilm removal and microbial viability [2]. In most cases, no ideal method or model exists that works perfectly in all circumstances and provides all the answers. Therefore, a combination of two or more complementary methods may need to use, and their strengths and weaknesses need to be considered when interpreting the results [25]. Therefore, in the present study, two types of multispecies root canal biofilm models were established in combination with two assessment methods, SEM and CLSM, with the aim to obtain more meaningful results.

In recent years, many studies have been conducted on the performance of Er:YAG LAI techniques in endodontic irrigation. However, a consensus has yet to be reached regarding the antibiofilm efficacy of Er:YAG LAI techniques (e.g., PIPS and SWEEPS) in comparison to ultrasonic-activated irrigation (e.g., PUI) [13, 28]. While some studies have demonstrated the superior performance of Er:YAG LAI techniques [6, 10, 29, 30], others have found no significant difference [31, 32]. In our study, Er:YAG LAI techniques exhibited better antibiofilm results than CNI and PUI in both Model 1 and Model 2, especially in the hard-to-reach areas. As a result, the null hypothesis of the present study was rejected. The heightened effectiveness of Er:YAG LAI techniques could be attributed to their distinctive mechanism of action [33–35]. They utilize shock wave-enhanced emission photoacoustic streaming, which induces rapid fluid motion within the root canal. This dynamic fluid movement ensures comprehensive removal of bacterial biofilms, even from the most intricate regions of the canal system, such as fins and dentinal tubules. Furthermore, the Er:YAG LAI techniques augment the penetration and efficacy of irrigating solutions within dentinal tubules [33–35]. It is important to underline those techniques such as CNI, PUI, and EDDY necessitate the insertion and activation of their tips near the difficult-to-access areas within the canal. In contrast, LAI operates differently; its effectiveness is achieved with the laser tip positioned at the entrance of the canal, making its impact remote and not dependent on the precise placement of the tip inside the canal [6].

The effectiveness of EDDY in comparison to PUI for biofilm removal has been a subject of debate. Some studies have suggested that EDDY is more effective in targeting microbes than PUI [36, 37], while other research has found no significant differences between the two methods [6, 12]. In our study, EDDY demonstrated a superior ability to reduce bacterial biofilm abundance compared to PUI in both experimental models. Several factors might explain this observation. Despite EDDY’s lower oscillation frequency compared to PUI, its displacement amplitude of 350 µm is significantly greater than PUI’s 75 µm [6]. High-speed imaging at 100,000 frames per second revealed that the EDDY tip executes three-dimensional orbital movements, in contrast to the primarily one-dimensional oscillation of the ultrasonic file and fluid flow [6, 14]. The EDDY tip’s motion generates oscillatory fluid dynamics within the root canal, potentially inducing shear stress against the canal walls, characterized by high velocities and energies [37]. Additionally, the vertical movement of the EDDY tip may enhance fluid dynamics, unlike the stationary position of the ultrasonic file. The closed apex and open canal entrance of the root canal system likely contribute to an overlapping directional flow [37]. The findings from this study suggest that EDDY is particularly effective in removing biofilm bacteria from challenging locations, such as fins and dentinal tubules.

The discrepancies between the results of the current study and previous studies may be attributed to differences in methodological design, including variations in root canal anatomy, the type of bacteria or biofilm incubated, the duration of bacterial incubation, the instrumentation protocol, the irrigation solution used, and the irrigation method parameters. The experimental setups in this study, which involved cultivating mature multispecies biofilms in apical uninstrumented areas and within dentinal tubules, presented greater challenges than those typically encountered in other studies. In Model 1, all five irrigation techniques demonstrated effective biofilm reduction in the main canal space (outside the groove), with SWEEPS, PIPS, and EDDY achieving the highest and nearly identical biofilm removal rates of 99.56%, 99.46%, and 99.46% respectively. This was closely followed by PUI with 99.12% and CNI with 98.34%. The minor differences among the techniques in the easily accessible area in this study may shed light on the disparate outcomes seen in previous research.

In our study, the SWEEPS group outperformed the PIPS group in treating multispecies biofilms within apical root canals and dentinal tubules. This study is the first to compare PIPS and SWEEPS in terms of treating multispecies biofilms in uninstrumented areas and dentinal tubules. Therefore, our findings could not be compared with those of other reports. PIPS and SWEEPS are both Er:YAG LAI techniques used in endodontics for root canal cleaning and disinfection. SWEEPS is a more recent Er:YAG LAI technique introduced to improve the cleaning and disinfecting efficacy of PIPS. While PIPS relies on a single laser pulse to heat irrigants, creating vapor bubbles that produce a cavitation effect for cleaning, SWEEPS advances this method by employing two consecutive laser pulses [19, 38]. The second pulse in SWEEPS is timed to coincide with the final phase of the initial bubble’s collapse, accelerating its collapse and emitting shock waves [19, 38]. These shock waves enhance cleaning efficacy, especially in narrow root canals, making SWEEPS a more optimized technique compared to PIPS [19, 38].

In this study, the Er:YAG LAI techniques, SWEEPS and PIPS, along with EDDY, exhibited notable efficacy in biofilm removal and bacterial killing within hard-to-reach areas, such as inside the groove and dentinal tubules. A recent investigation [24] assessed the effectiveness of SWEEPS, EDDY, and CNI in eliminating biofilm-like hydrogel from isthmuses, examining the impact of isthmus morphology on debridement efficacy and the physical mechanisms underlying SWEEPS and EDDY activation in these areas. This research may provide insights into the effectiveness of Er:YAG LAI techniques and EDDY in addressing biofilms situated in challenging locations. High-speed imaging revealed that EDDY generated eddies and stable cavitation within the isthmus, whereas SWEEPS induced transient cavitation and pulsed horizontal flow. In closed isthmuses, SWEEPS achieved greater maximum particle speed and more efficient biofilm removal compared to EDDY. The closed isthmuses, particularly those that are short and narrow (0.15 mm wide), bear resemblance to the grooves examined in our research. Interestingly, compared to PIPS, EDDY showed a reduced ability to remove biofilms from apical artificial grooves. However, EDDY exhibited superior bacterial killing efficacy within the dentinal tubules. This disparity could be attributed to the distinct irrigation mechanisms. EDDY employs a flexible polyamide tip that moves in a three-dimensional pattern with high amplitude oscillations [14]. This oscillation induces hydrodynamic phenomena, such as cavitation and microstreaming. These effects enhance the penetration of NaOCl into the dentinal tubules. On the other hand, PIPS utilizes laser energy to activate irrigants within the root canal. Upon firing the laser, rapid photoacoustic waves are generated, leading to the formation of minuscule vapor bubbles [19]. The subsequent expansion and collapse of these bubbles exert powerful streaming forces, propelling NaOCl throughout the canal system. This mechanism enables PIPS to access and clean areas of the canal system that are typically challenging to reach; thus, PIPS is particularly effective at removing biofilms from artificial grooves [39].

While a large number of in vitro studies have focused on comparing the performance of Er:YAG LAI techniques with other methods in endodontic irrigation, there are limited clinical reports on the effect of Er:YAG LAI techniques on postoperative pain following root canal treatment (RCT) [40–42] and the success rate of RCT [43]. A randomized clinical study by Dagher et al. reported that PIPS was as effective as CNI in relation to postoperative pain [40]. Moreover, a randomized clinical study by Erkan et al. revealed that PIPS and SWEEPS resulted in lower postoperative pain scores and levels than other activation systems, including EDDY, PUI, and manual dynamic activation [41]. Interestingly, another randomized clinical study by Mandras et al. assessed the effect of PIPS, compared to CNI, on reducing the root canal system bacterial count and on postoperative patient quality of life (QoL) after RCT [42]. The findings of this study indicated that PIPS was more effective at reducing bacterial counts and achieved better QoL indicators in the days immediately following RCT, including reduced maximum pain, eating difficulties, and challenges in performing daily functions. To the best of our knowledge, there is only one published randomized controlled trial evaluating the effect of CNI, PUI, and LAI on the clinical and radiographic success rate of RCT [43]. In this study, PUI and LAI achieved similar but greater success rates than CNI at the 12-month follow-up. Notably, this study evaluated only the effect of LAI on the short-term outcome of RCT.

This laboratory-based study has several limitations. Infection of the Model 2 samples was similar to that of the dentin block model, in which bacteria were forced into the dentinal tubules by centrifugation to produce a more standardized deep infection [44]. This method of contamination differs from the way root canals are infected in vivo, and centrifugation process potentially has a negative impact on the bacteria [25, 44]. In our pilot experiments, some untreated control samples were Gram stained using the MycoLight™ Rapid Fluorescence Bacterial Gram Stain Kit (A22413, AAT Bioquest, Pleasanton, CA, USA) to investigate the composition of bacteria that eventually invade and grow in dentinal tubules. The CLSM images confirmed the deep penetration and heavy infection of both gram-positive (G +) and gram-negative (G-) bacteria. However, the exact species of bacteria involved remain unknown. Another limitation is that the teeth used to establish the biofilm models were basically young teeth (extracted for orthodontic purposes) to avoid sclerotic dentin; therefore, the findings may not be suitable for direct extrapolation to old human root canals due to the high possibility of sclerotic dentin in the apical root. Furthermore, during the CLSM analysis, it was observed that the images associated with the SWEEPS, PIPS, and EDDY groups consistently exhibited significantly lower brightness, indicative of reduced fluorescence intensity. This suggests that the three irrigation techniques were effective not only in eradicating bacteria but also in dislodging them from the dentinal tubules. Unfortunately, a robust method to quantitatively assess this removal has yet to be identified. Despite these challenges, Model 2 introduces a novel approach for assessing the efficacy of antimicrobial treatments.

## Conclusion

Within the limitations of this in vitro study, both Er:YAG LAI techniques and EDDY demonstrated significant efficacy in removing biofilms from apical artificial grooves and killing bacteria colonized in the dentinal tubules, areas that are typically challenging to access. Further high-quality pre-clinical studies and clinical trials with larger sample sizes are needed to evaluate the effectiveness of Er:YAG LAI techniques for the long-term success of RCT.

## Data Availability

The corresponding author can provide the datasets used and/or analyzed during the current study upon reasonable request.

## References

[1] Bordea IR, Hanna R, Chiniforush N, Grădinaru E, Câmpian RS, Sîrbu A, Amaroli A, Benedicenti S (2020). Evaluation of the outcome of various laser therapy applications in root canal disinfection: a systematic review. Photodiagnosis Photodyn Ther.

[2] Swimberghe RCD, Coenye T, De Moor RJG, Meire MA (2019). Biofilm model systems for root canal disinfection: a literature review. Int Endod J.

[3] Svensäter G, Bergenholtz G (2004). Biofilms in endodontic infections. Endod Top.

[4] Nair PN, Henry S, Cano V, Vera J (2005). Microbial status of apical root canal system of human mandibular first molars with primary apical periodontitis after “one-visit” endodontic treatment. Oral Surg Oral Med Oral Pathol Oral Radiol Endod.

[5] Nair PNR (2004). Pathogenesis of apical periodontitis and the causes of endodontic failures. Crit Rev Oral Biol Med.

[6] Swimberghe RCD, De Clercq A, De Moor RJG, Meire MA (2019). Efficacy of sonically, ultrasonically and laser-activated irrigation in removing a biofilm- mimicking hydrogel from an isthmus model. Int Endod J.

[7] Haapasalo M, Shen Y, Wang Z, Gao Y (2014). Irrigation in endodontics. Br Dent J.

[8] Boutsioukis C, Arias-Moliz MT (2022). Present status and future directions - irrigants and irrigation methods. Int Endod J.

[9] Liu H, Nio S, Shen Y. Sodium hypochlorite against Enterococcus faecalis biofilm in dentinal tubules: effect of concentration, temperature, and exposure time. Odontology. 2023;30. 10.1007/s10266-023-00850-9. 10.1007/s10266-023-00850-9 37646916

[10] Kumar K, Teoh YY, Walsh LJ (2023). Root canal cleaning in roots with complex canals using agitated irrigation fluids. Aust Endod J.

[11] Bao P, Shen Y, Lin J, Haapasalo M (2017). In vitro efficacy of XP-endo Finisher with 2 different protocols on biofilm removal from apical root canals. J Endod.

[12] Al-Zuhair H, Su Z, Liu H, Wang Z, Haapasalo M, Hieawy A, Gao Y, Shen Y (2023). Antimicrobial effects of agitational irrigation on single- and multi-species biofilms in dentin canals. Odontology.

[13] Josic U, Mazzitelli C, Maravic T, Fidler A, Breschi L, Mazzoni A (2022). Biofilm in endodontics: In vitro cultivation possibilities, sonic-, ultrasonic- and laser-assisted removal techniques and evaluation of the cleaning efficacy. Polymers (Basel).

[14] Chu X, Feng S, Zhou W, Xu S, Zeng X (2023). Cleaning efficacy of EDDY versus ultrasonically-activated irrigation in root canals: a systematic review and meta-analysis. BMC Oral Health.

[15] Liu H, Shen Y, Haapasalo M (2023). Effectiveness of six irrigation techniques with sodium hypochlorite in tissue dissolution. Cureus.

[16] Yang Q, Liu MW, Zhu LX, Peng B (2020). Micro-CT study on the removal of accumulated hard-tissue debris from the root canal system of mandibular molars when using a novel laser-activated irrigation approach. Int Endod J.

[17] DiVito E, Peters OA, Olivi G (2012). Effectiveness of the erbium:YAG laser and new design radial and stripped tips in removing the smear layer after root canal instrumentation. Lasers Med Sci.

[18] Bago I, Batelja-Vuletić L, Tarle A, Sesar A, Anić I (2022). Novel laser activated photoacoustic streaming for removing pulp remnants from round root canals after single file reciprocating instrumentation. Photodiagnosis Photodyn Ther.

[19] Mancini M, Cerroni L, Palopoli P, Olivi G, Olivi M, Buoni C, Cianconi L (2021). FESEM evaluation of smear layer removal from conservatively shaped canals: laser activated irrigation (PIPS and SWEEPS) compared to sonic and passive ultrasonic activation-an ex vivo study. BMC Oral Health.

[20] Vatanpour M, Toursavadkouhi S, Sajjad S (2022). Comparison of three irrigation methods: SWEEPS, ultrasonic, and traditional irrigation, in smear layer and debris removal abilities in the root canal, beyond the fractured instrument. Photodiagnosis Photodyn Ther.

[21] Ensafi F, Fazlyab M, Chiniforush N, Akhavan H (2022). Comparative effects of SWEEPS technique and antimicrobial photodynamic therapy by using curcumin and nano-curcumin on Enterococcus faecalis biofilm in root canal treatment. Photodiagnosis Photodyn Ther.

[22] Lei L, Wang F, Wang Y, Li Y, Huang X (2022). Laser activated irrigation with SWEEPS modality reduces concentration of sodium hypochlorite in root canal irrigation. Photodiagnosis Photodyn Ther.

[23] Wen C, Yan L, Kong Y, Zhao J, Li Y, Jiang Q (2021). The antibacterial efficacy of photon-initiated photoacoustic streaming in root canals with different diameters or tapers. BMC Oral Health.

[24] Robberecht L, Delattre J, Meire M (2023). Isthmus morphology influences debridement efficacy of activated irrigation: a laboratory study involving biofilm mimicking hydrogel removal and high-speed imaging. Int Endod J.

[25] Boutsioukis C, Arias-Moliz MT, Chávez de Paz LE (2022). A critical analysis of research methods and experimental models to study irrigants and irrigation systems. Int Endod J.

[26] Nagendrababu V, Murray PE, Ordinola-Zapata R, Peters OA, Rôças IN, Siqueira JF, Priya E, Jayaraman J, J Pulikkotil S, Camilleri J, Boutsioukis C, Rossi-Fedele G, Dummer PMH (2021). PRILE 2021 guidelines for reporting laboratory studies in Endodontology: a consensus-based development. Int Endod J.

[27] Shen Y, Qian W, Chung C, Olsen I, Haapasalo M (2009). Evaluation of the effect of two chlorhexidine preparations on biofilm bacteria in vitro: a three-dimensional quantitative analysis. J Endod.

[28] Badami V, Akarapu S, Kethineni H, Mittapalli SP, Bala KR, Fatima SF (2023). Efficacy of laser-activated irrigation versus ultrasonic-activated irrigation: a systematic review. Cureus.

[29] Ordinola-Zapata R, Bramante CM, Aprecio RM, Handysides R, Jaramillo DE (2014). Biofilm removal by 6% sodium hypochlorite activated by different irrigation techniques. Int Endod J.

[30] Neelakantan P, Cheng CQ, Mohanraj R, Sriraman P, Subbarao C, Sharma S (2015). Antibiofilm activity of three irrigation protocols activated by ultrasonic, diode laser or Er:YAG laser in vitro. Int Endod J.

[31] Seghayer I, Lee AHC, Cheung GSP, Zhang C (2023). Effect of passive ultrasonic irrigation, Er, Cr:YSGG laser, and photon-induced photoacoustic streaming against enterococcus faecalis biofilms in the apical third of root canals. Bioengineering (Basel).

[32] Akdere SK, Aydin ZU, Erdönmez D (2023). Antimicrobial effectiveness of different irrigation activation techniques on teeth with artificial internal root resorption and contaminated with Enterococcus faecalis: a confocal laser scanning, icroscopy analysis. Lasers Med Sci.

[33] Galler KM, Grubmüller V, Schlichting R, Widbiller M, Eidt A, Schuller C, Wölflick M, Hiller KA, Buchalla W (2019). Penetration depth of irrigants into root dentine after sonic, ultrasonic and photoacoustic activation. Int Endod J.

[34] Kosarieh E, Bolhari B, Sanjari Pirayvatlou S, Kharazifard MJ, Sattari Khavas S, Jafarnia S, Saberi S (2021). Effect of Er:YAG laser irradiation using SWEEPS and PIPS technique on dye penetration depth after root canal preparation. Photodiagnosis Photodyn Ther.

[35] Akcay M, Arslan H, Mese M, Durmus N, Capar ID (2017). Effect of photon-initiated photoacoustic streaming, passive ultrasonic, and sonic irrigation techniques on dentinal tubule penetration of irrigation solution: a confocal microscopic study. Clin Oral Investig.

[36] Hoedke D, Kaulika N, Dommisch H, Schlafer S, Shemesh H, Bitter K (2021). Reduction of dual-species biofilm after sonic- or ultrasonic-activated irrigation protocols: a laboratory study. Int Endod J.

[37] Neuhaus KW, Liebi M, Stauffacher S, Eick S, Lussi A (2016). Antibacterial efficacy of a new sonic irrigation device for root canal disinfection. J Endod.

[38] Lukač N, Jezeršek M (2018). Amplification of pressure waves in laser-assisted endodontics with synchronized delivery of Er:YAG laser pulses. Lasers Med Sci.

[39] Yargici VH, Kaptan RF (2022). Evaluation of debris removal efficacy of conventional syringe, Irrisafe, XP-endo Finisher file, and photon-induced photoacoustic-streaming methods in teeth with artificial internal resorption using two different methodologies. Photobiomodul Photomed Laser Surg.

[40] Dagher J, El Feghali R, Parker S, Benedicenti S, Zogheib C (2019). Postoperative quality of life following conventional endodontic intracanal irrigation compared with laser-activated irrigation: a randomized clinical study. Photobiomodul Photomed Laser Surg.

[41] Erkan E, Gündoğar M, Uslu G, Özyürek T (2022). Postoperative pain after SWEEPS, PIPS, sonic and ultrasonic-assisted irrigation activation techniques: a randomized clinical trial. Odontology.

[42] Mandras N, Pasqualini D, Roana J, Tullio V, Banche G, Gianello E, Bonino F, Cuffini AM, Berutti E, Alovisi M (2020). Influence of photon-induced photoacoustic streaming (PIPS) on root canal disinfection and post-operative pain: A randomized clinical trial. J Clin Med.

[43] Verma A, Yadav RK, Tikku AP, Chandra A, Verma P, Bharti R, Shakya VK (2020). A randomized controlled trial of endodontic treatment using ultrasonic irrigation and laser activated irrigation to evaluate healing in chronic apical periodontitis. J Clin Exp Dent.

[44] Ma J, Wang Z, Shen Y, Haapasalo M (2011). A new noninvasive model to study the effectiveness of dentin disinfection by using confocal laser scanning microscopy. J Endod.

